# Differentially Severe Cognitive Effects of Compromised Cerebral Blood Flow in Aged Mice: Association with Myelin Degradation and Microglia Activation

**DOI:** 10.3389/fnagi.2017.00191

**Published:** 2017-06-16

**Authors:** Gilly Wolf, Amit Lotan, Tzuri Lifschytz, Hagar Ben-Ari, Tirzah Kreisel Merzel, Pavel Tatarskyy, Michael Valitzky, Ben Mernick, Elad Avidan, Nickolay Koroukhov, Bernard Lerer

**Affiliations:** ^1^Biological Psychiatry Laboratory, Hadassah-Hebrew University Medical CenterJerusalem, Israel; ^2^Hadassah BrainLabs—National Knowledge Center for Research on Brain DiseasesJerusalem, Israel; ^3^Departments of Psychology and Life Sciences, School of Sciences, Achva Academic CollegeBe’er Tuvia, Israel; ^4^Department of Developmental Biology and Cancer Research, Hadassah-Hebrew University Medical SchoolJerusalem, Israel; ^5^Neurology Laboratory, Department of Neurology, Hadassah-Hebrew University Medical CenterJerusalem, Israel; ^6^Developmental Psychopathology Laboratory, Department of Psychology, University of HaifaHaifa, Israel; ^7^Cardiovascular Research Center, Hadassah-Hebrew University Medical CenterJerusalem, Israel

**Keywords:** bilateral carotid artery stenosis, vascular dementia, aging, cerebral hypoperfusion, mouse model, white matter lesions, microglia

## Abstract

Bilateral common carotid artery stenosis (BCAS) models the effects of compromised cerebral blood flow on brain structure and function in mice. We compared the effects of BCAS in aged (21 month) and young adult (3 month) female mice, anticipating a differentially more severe effect in the older mice. Four weeks after surgery there was a significant age by time by treatment interaction on the radial-arm water maze (RAWM; *p* = 0.014): on the first day of the test, latencies of old mice were longer compared to the latencies of young adult mice, independent of BCAS. However, on the second day of the test, latencies of old BCAS mice were significantly longer than old control mice (*p* = 0.049), while latencies of old controls were similar to those of the young adult mice, indicating more severe impairment of hippocampal dependent learning and working memory by BCAS in the older mice. Fluorescence staining of myelin basic protein (MBP) showed that old age and BCAS both induced a significant decrease in fluorescence intensity. Evaluation of the number oligodendrocyte precursor cells demonstrated augmented myelin replacement in old BCAS mice (*p* < 0.05) compared with young adult BCAS and old control mice. While microglia morphology was assessed as normal in young adult control and young adult BCAS mice, microglia of old BCAS mice exhibited striking activation in the area of degraded myelin compared to young adult BCAS (*p* < 0.01) and old control mice (*p* < 0.05). These findings show a differentially more severe effect of cerebral hypoperfusion on cognitive function, myelin integrity and inflammatory processes in aged mice. Hypoperfusion may exacerbate degradation initiated by aging, which may induce more severe neuronal and cognitive phenotypes.

## Introduction

Improvements in public health have led to a dramatic increase in the human lifespan over the last century (Vaupel, 2010). This has been accompanied by increased risk for the development of neurodegenerative and neuropsychiatric disorders of late life, including cognitive impairment, dementia and depression (Beach et al., 2007; Roher et al., 2011; Kaup et al., 2016). After Alzheimer’s disease, cerebrovascular disease is the second most common cause of age-related cognitive impairment and dementia (O’Brien and Thomas, 2015; Kalaria, 2016). Perfusion deficits have been shown to contribute to depressive behavior among the elderly (Popa-Wagner et al., 2014). Vascular cognitive impairment (VCI) is characterized as a neurocognitive disorder but also incorporates behavioral symptoms, locomotor abnormalities and autonomic dysfunction. Although a definitive pathological definition of VCI is lacking, it is generally accepted that cerebral vessel disease, including cerebral atherosclerosis and arteriolosclerosis, results in global and/or local cerebral hypoperfusion leading to cortical and subcortical infarcts (Brun, 1994; Kalaria, 2016). White matter lesions (WMLs) represent pathologically incomplete infarcts in white matter, are common in cerebral hypoperfusion states and are evident as white matter hyperintensities on MRI (Fernando et al., 2006).

There are no specific treatments for VCI. Progress has been difficult because of uncertainties over disease classification and diagnostic criteria, controversy as to the exact relationship between cerebrovascular pathology and cognitive impairment and a paucity of tractable treatment targets. The investigation of relevant animal models has proven very valuable in exploring the pathogenesis of cognitive and affective symptoms induced by cerebral hypoperfusion. A mouse model of chronic cerebral hypoperfusion, in which the cerebral white matter is damaged without significant gray matter lesions, is particularly relevant (Shibata et al., 2004). The WMLs in this model are induced by bilateral carotid artery stenosis (BCAS). The model has demonstrated good reproducibility of WMLs characterized by blood-brain barrier disruption, glial activation, oxidative stress and oligodendrocyte loss following chronic cerebral hypoperfusion (Ihara and Tomimoto, 2011). BCAS provides a powerful tool to study the impact of WMLs on cognition. Working memory deficits, attributable to damage of frontal-subcortical circuits, were evident 30 days after BCAS on various spatial and reference memory paradigms (Shibata et al., 2007; Coltman et al., 2011). Holland et al. (2015) conducted MRI studies on BCAS operated mice and detected the presence of multiple hemorrhages, infarcts and white matter disruptions in these mice compared to sham operated mice. A recent review concluded that the BCAS hypoperfusion mouse model is probably the most suitable animal model for studying VCI (Bink et al., 2013).

To the best of our knowledge, employment of the BCAS model has been limited to young adult and middle-aged rodents and differential effects of cerebral hypoperfusion in young adult and aged mice have not been examined previously. VCI rates rise exponentially with age with risk roughly doubling every 5 years (Jorm and Jolley, 1998). Considering the robust alterations in biologic landscape and physiologic homeostasis that occur with aging (Finch and Tanzi, 1997), we suggest that animal models that take into account age-by-disease interaction, or performed on older animals, could have better face, construct and possibly predictive validity for the human disorder that they model. Such combined models have been successfully implemented in other neuropsychiatric disorders such as late-life depression (Erraji-Benchekroun et al., 2005; McKinney and Sibille, 2013). Indeed, several manipulations that may contribute to neurodegenerative disorders induce more severe outcomes in old compared with young adult animals. For example, Tournissac et al. (2016) demonstrated potentiated cold-induced tau phosphorylation in old mice, a mechanism that may contribute to the susceptibility of older populations to develop Alzheimer disease. Nigrostriatal dopaminergic neurons of older mice appeared more sensitive to neurotoxicity (Thiruchelvam et al., 2003) and to histamine-induced cataplexy and less responsive to L-DOPA (Ionov and Severtsev, 2012), which sheds further light on the mechanisms involved in Parkinson’s disease.

In the current study we examined the effect of BCAS on behavior and cognition in young adult and aged female mice and on relevant neurobiological outcomes such as neurogenesis, integrity of white matter and microglial activation. Our aim was to specifically characterize the effects of cerebral hypoperfusion induced by BCAS in aged mice and to identify cognitive and affective consequences of BCAS that are differentially manifested in aged mice and reflect a joint or interactive effect of age and impaired cerebral blood flow on the neural correlates of these behavioral phenomena.

## Materials and Methods

### Animals

Thirty-eight female C57BL/6JRccHsd mice aged 3 (young adult, *n* = 19) and 21 months (old, *n* = 19) were used in this study (Harlan Laboratory). Mice were housed in groups of 2–3 in 26.5 × 20 × 13.5 cm cages in the SPF-certified facility of the Sharett Institute, Hadassah-Hebrew University Medical Center. Food and water were provided *ad libitum*. Old and young adult mice were randomly allocated to equally sized subgroups that underwent BCAS microsurgery or a sham procedure (Control) before baseline. Mice were weighed twice weekly, starting 1 week prior to BCAS microsurgery. All measurements were performed during the light phase of a 12 h light-dark cycle (lights on at 07:00). Behavioral tests (see below) were performed in an SPF-certified examination room in our laboratory. The experimental procedure was according to the ARRIVE guidelines (Kilkenny et al., 2012) and NIH approval number: OPRR-A01-5011. The experiments were approved by the Hebrew University Ethics Committee on Animal Care and Use (Applications MD-14-14015-4; MD-16-14679-4) As an AAALAC-accredited institute the Hebrew University Ethics Committee follows the NRC Guide for the Care and Use of Laboratory Animals.

### BCAS Microsurgery

Cerebral hypoperfusion was induced by means of 0.18 mm internal diameter steel coils that were implanted around both common carotid arteries followed by a 4-week hiatus before starting the behavioral experiments. Coils of this diameter were previously demonstrated to induce a 25%–33% decrease in cerebral blood flow and lesions in the white matter without involvement of gray matter (Shibata et al., 2004). On the day of the first surgery, the animals were brought into a SPF environment in the Center for Cardiac Research, Hadassah Medical Center and entered into an animal operation room. Each animal was first administered analgesia with Rimadyl 5 mg/kg i.p. (0.25 ml for 25 g animal body weight) and then anesthetized using a mixture of ketamine and xylazine (ketamine 70 mg per kg animal body weight, xylazine 10 mg per kg animal body weight, mixture administered 0.25 ml per 25 g animal body weight). After the animal was anesthetized, the upper chest and neck area were shaved and disinfected with chlorhexidine solution and then a longitudinal section was made with a surgical blade at the right side of the neck, and the underlying fascia was separated, exposing the muscles below. The sternocleidomastoid muscle was deflected, and the common carotid artery was exposed. Two Ethicon sutures 4-0 were inserted below the exposed part of the artery and the specialized constricting coil was wrapped around the part of the artery between the two sutures. The coil is produced by Wuxi Samini Spring Co., Jinagsu, China. It is made of gold plated piano spring stainless steel, with 0.18 mm internal diameter and is 2.5 mm total length. After the coil implantation, the two silk sutures were removed, and the cut was sutured. Sham operated animals underwent the same procedure but no coil was implanted.

**Figure 1:**
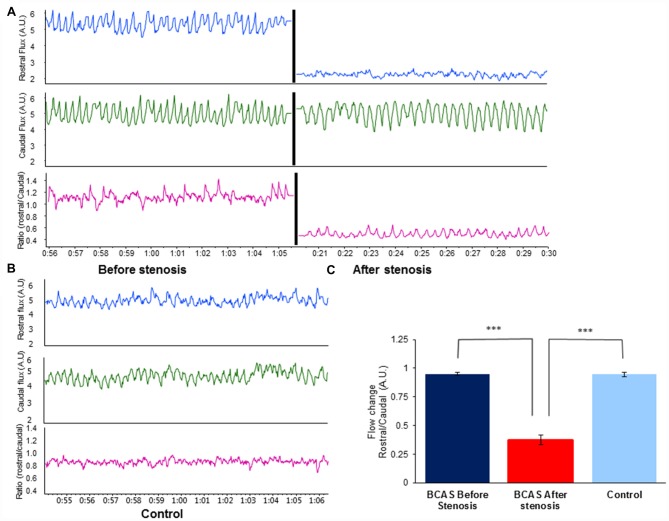
Effect of bilateral common carotid artery stenosis (BCAS) microsurgery on changes in common carotid artery flood flow. **(A)** External carotid blood flow was recorded at a sample rate of 40/s with a laser Doppler flowmeter. Top: rostral flux measurement before (left) and after stenosis (right) demonstrates decrease in blood flow. Middle: caudal flux measurement before (left) and after stenosis (right). No change in blood flow was evident. Bottom: ratio of rostral to caudal flux in arbitrary units (A.U.) before (left) and after stenosis (right). **(B)** Record of external carotid blood flow in sham-operated control mouse: rostral flux (top), caudal flux (middle) and ratio of rostral to caudal flux in A.U. (bottom). **(C)** Flow change (A.U.) ratio of rostral/caudal measurement revealed a decrease of ~70% in blood blow before and after BCAS microsurgery (*t*_(4)_ = −18.401, *p* < 0.00001), and compared with sham-operated mice (*t*_(8)_ = −12.675, *p* < 0.00001; ***p < 0.00001).

To validate hypoperfusion, external carotid blood flow was recorded at a sample rate of 40/s with a laser Doppler flowmeter (DRT4, Moor, Devon, UK) in separate group of mice. A standardized protocol was used for the flux measurements procedure. Two blunt needle end delivery laser Doppler probes (VP4s, stainless steel tube 26-gauge needle, external diameter 0.46 mm, Moor, Devon, UK), were placed at fixed distance between each other, one rostral and one caudal to the coil, and at fixed location in each mouse (*n* = 5 per group). Flux was measured before and after coil placement. Data were recorded (1 KHz) using a Power Lab 16/35 data acquisition system (AD instruments, UK) and the data acquisition software LabChart 8.1.5 software (AD Instruments). Blood flow was extrapolated each 100 ms. Control blood flow change was evaluated as the ratio between the rostral and caudal measurement. The same ratio (rostral/caudal Flux) was measured before and after micro coil placement. The blood flow change was compared to the pre-stenotic state and to the sham-operated group. Figure 1 presents the rostral/caudal ratio of mice before and after coil placement, as well as the ratio of sham-operated mice. As can be seen, rostral flux decreased by ~70% following coil placement compared with rostral flux prior to coil placement (*t*_(4)_ = −18.401, *p* < 0.00001), and with rostral flux of sham-operated mice (*t*_(8)_ = −12.675, *p* < 0.00001). No such change was noticed in sham-operated mice.

**Figure 2:**
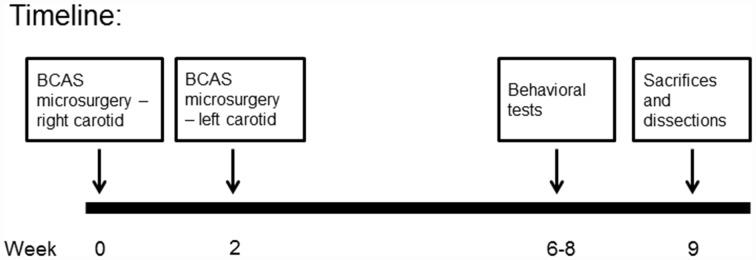
Visualization of the experimental timeline.

Following implantation, operated animals were given another i.p. injection of Rimadyl, and were monitored until they regained consciousness. After that the animals were returned to their home cages and were followed up for the next 3 days after surgery. Two weeks after the first coil implantation surgery, the surgical procedure was repeated (for both coil implanted and sham operated animals) for the untreated common carotid artery, with post operational recovery and follow-up similar to the first operation. Preliminary surgeries performed in our laboratory indicated that this 2-week break is necessary for the survival of the old mice. Figure 2 provides a description of the experimental timeline.

In order to prevent unnecessary suffering, mice that lost at least 20% of their initial weight or 10% between two weighings were to be excluded. Mice that spontaneously developed tumors (as sometimes happen in old age) were to be excluded as well. None of the mice met the weight loss criteria; Tumors were observed in four old sham mice and one old BCAS mouse which were excluded; one old BCAS mouse died shortly after microsurgery. Ten young adult mice were allocated to BCAS microsurgery (all survived) and 10 old mice (eight survived). Of the nine young adult mice that underwent sham surgery, all survived; of the nine old mice, five survived.

### Behavioral—Cognitive Tests

Mice underwent a 3-week behavioral and cognitive test battery. Behavioral tests started 4 weeks following BCAS microsurgery (see Figure 2). All behavioral tests were recorded and analyzed using the Ethovision 10 system. All surviving mice were included in the behavioral tests. Mice per group ranged from 5 to 10, an acceptable n that is frequently used in studies in which the BCAS model is employed.

#### Social Exploration (SE) Test

Mice were individually placed in the arena for 15 min habituation, then presented with a young (~3 week old) mouse (juvenile) for 2 min. The amount of time mice spent sniffing the juvenile was measured. Social exploration (SE) was tested prior to microsurgeries (baseline) as well as during the 3rd week of the behavioral battery. The researchers were blind to the experimental conditions.

#### Open Field (OF) Test

Mice were placed in a 50 × 50 cm arena surrounded by 40 cm walls for a 6 min test. The center of the arena was defined as a 25 × 25 square in the middle of the arena. Velocity of movement and presence in the center of the arena were measured. Open field (OF) test was performed during the first week of the behavioral battery, using Ethovision 10 system, providing fully computerized, blinded and unbiased measurement.

#### Radial-Arm Water Maze (RAWM)

A 2-day protocol of the radial-arm water maze (RAWM) test was used (Alamed et al., 2006). On day 1, mice were trained with 12 trials alternating between visible and invisible platform, followed by three test trials with invisible platform. On day 2 mice had 15 trials with the invisible platform. Latency to the platform on trials 13–15 was calculated. RAWM test was performed during the 3rd week of the behavioral battery, using the Ethovision 10 system, providing fully computerized, blinded and unbiased measurement.

#### Novel Object Recognition (NOR)

This test consisted of two parts—sample object exposure and novel object test. For sample object exposure mice were placed in a 25 × 25 cm arena, containing two identical objects for 10 min and then returned to their home cage. The novel object test took place 1 h following the sample object exposure. Mice were returned to the arena, which contained the sample object and a novel object for 4 min. The ratio of exploration of the novel object and the total exploration of the two objects were calculated. Novel object recognition (NOR) test was performed during the 2nd week of the behavioral battery, using Ethovision 10 system, providing fully computerized, blinded and unbiased measurement.

### Immunohistochemistry

Animals were perfused transcardially with cold phosphate-buffered saline (PBS) followed by 4% formaldehyde and the brains were quickly removed and placed in 4% formaldehyde. After 24 h, the brains were placed in 20% sucrose solution in DDW and then frozen at optimal cutting temperature. Brains were dissected to 50 μm frozen floating sections.

Sections were taken from the area of the frontal cortex, +2.1 to +1.42 (relative to bregma). Five sections were analyzed for each mouse. Mice per group ranged from two to six, dependent on the available specimens. Due to the small n, that is susceptible to type I (false positive) and type II (false negative) errors, we treated these results cautiously and regard them as preliminary.

#### Myelin Basic Protein (MBP) Immunofluorescence Staining

Myelin basic protein (MBP) staining was performed on 5 mm frozen floating brain sections. The sections were fixed in methanol, washed twice with PBS and incubated overnight in 1% bovine serum and 0.1% triton in 1xPBS with the primary antibody (Anti- MBP 1:100, Millipore-Temecula, CA, USA) at 4°C. Sections were then incubated with the secondary antibody (Cy3, 1:400; Jackson, ImmunoResearch) for 2 h at room temperature (RT) and counter-stained with DAPI (Sigma, Israel).

#### Microglia Visualization

Microglia were visualized using an antibody to the microglia activation marker ionized calcium-binding adapter molecule-1 (Iba-1). Brain sections were fixed in methanol. After three PBS washes they were incubated overnight in 1% bovine serum and 0.1% triton in 1xPBS with the primary antibody (rabbit anti Iba-1 1:200, Wako, Osaka, Japan) at 4°C. Sections were then incubated with the secondary antibody (Alexa Flour 488, Cy2-donkey anti rabbit, 1:200; Jackson, ImmunoResearch) for 1 h at RT and counter-stained with DAPI (Sigma, Israel).

#### Oligodendrocyte Visualization

Oligodendrocyte precursor cells were visualized using NG2 Chondroitin Sulfate proteoglycan marker. Brain sections were fixed in methanol. After three PBS washes they were incubated overnight in 1% bovine serum and 0.1% triton in 1xPBS with the primary antibody (Anti NG2 Chondroitin Sulfate proteoglycan 1:200, Millipore-Temecula, CA, USA) at 4°C. Sections were then incubated with the secondary antibody (Cy2, 1:400; Jackson, ImmunoResearch) for 2 h at RT and counter-stained with DAPI (Sigma, Israel).

MBP, microglia and oligodendrocyte images were captured using a Nikon DS-Qi2 microscope and camera and Olympus FV-1000 confocal microscope and camera (Tokyo, Japan). Images were analyzed with NIS-Elements AR software (NIS-Elements, Melville, NY, USA), Image Pro- Plus software (Media Cybernetics, Inc., Washington Rockville, MD, USA) and Olympus Fluoview software. MBP intensity was measured twice by an observer blind to the age and exposure. Microglial activation was determined by measuring soma (cell body) area and branch length using ×40 magnification. This analysis was performed on 5–10 randomly selected cells/section. In addition, microglia cells were counted in a defined area of the anterior commissure using ×20 magnification. All assessments were done by an observer blind to age and exposure.

All histological staining and visualization were performed by a researcher who was blind to the experimental conditions of the specimens. The experimental condition of each specimen was exposed only during analysis.

### Statistical Analysis

Data were analyzed using SPSS 19. Two-way analysis of variance (ANOVA) was performed, with repeated measures when appropriate, followed by univariate tests of simple main effects. Data in figures are given as mean ± standard error of the mean (SEM). *P* values < 0.05, two tailed, were regarded as statistically significant.

## Results

### Body Weight Change of Young Adult and Old Mice

**Figure 3:**
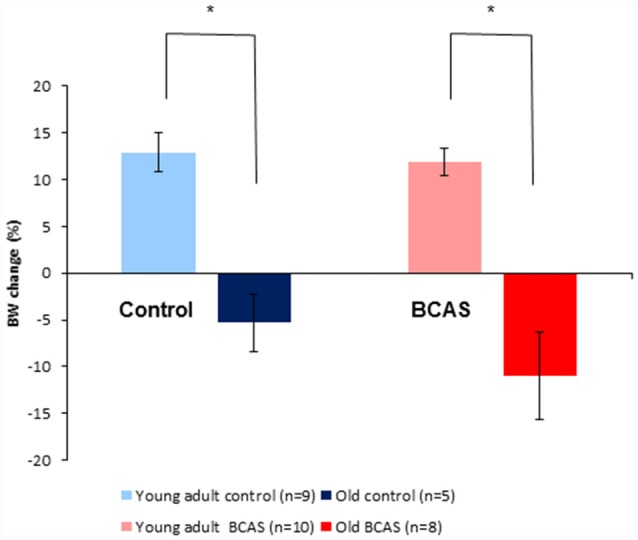
Effect of BCAS microsurgery on body weight in young adult and old mice. Change in body weight from baseline to the end of the 8th week following BCAS is shown. Old mice lost weight significantly, whereas young adult mice gained weight during the experimental period; two-way analysis of variance (ANOVA) with repeated measures revealed a significant age by time interaction (*F*_(1,28)_ = 42.75, *p* = 4 × 10^−7^; *p < 0.05).

As shown in Figure 3, old mice lost weight significantly whereas young adult mice gained weight during the experimental period. Two way ANOVA with repeated measures comparing change in body weight from baseline (i.e., before BCAS microsurgery) until 8 weeks post-surgery revealed a significant age by time interaction (*F*_(1,28)_ = 42.753, *p* = 4 × 10^−7^). Consistent with the finding of the main analysis, univariate tests of simple main effects were performed, revealing significant weight loss of old control mice compared with young adult controls (*F*_(1,28)_ = 19.398, *p* = 0.0001) and significant weight loss of old BCAS mice compare with young adult BCAS (*F*_(1,28)_ = 37.895, *p* = 10^−5^).

### Effect of BCAS on Behavioral and Cognitive Measures in Young Adult and Old Mice

#### Social Interaction in the Social Exploration (SE) Test

**Figure 4:**
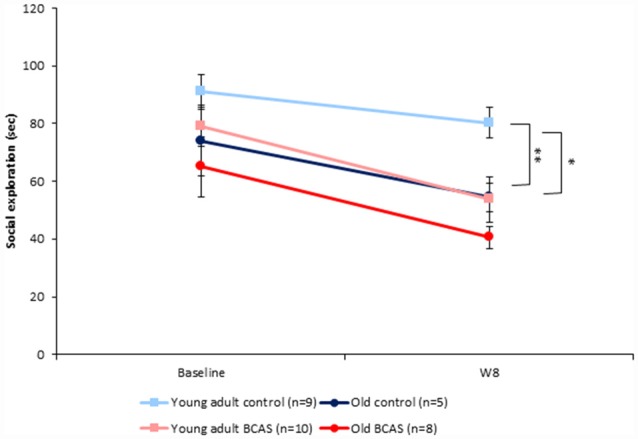
Effect of BCAS microsurgery on Social exploration (SE) of young adult and old mice. Both BCAS microsurgery and old age decreased the amount of time mice spent sniffing the juvenile. Two-way ANOVA with repeated measures comparing SE at baseline and 8 weeks following BCAS microsurgery revealed significant between-subject effects of age *F*_(1,29)_ = 10.561, *p* = 0.003) and treatment (*F*_(1,29)_ = 8.159, *p* = 0.008). No significant age by surgery interaction was found, indicating that the two factors reduced social interaction to a similar degree. Univariate tests of simple main effects revealed that young adult control mice displayed more exploratory behavior compared with young adult BCAS (*p* < 0.01) and old control mice (*p* < 0.05; *p < 0.05; **p < 0.01).

BCAS and old age both decreased the amount of time mice spent in social interaction. There were no significant differences between the groups at baseline as determined by ANOVA. Two-way ANOVA with repeated measures comparing SE at baseline and 8 weeks following BCAS microsurgery revealed significant between-subject effects of age (*F*_(1,29)_ = 10.561, *p* = 0.003) and treatment (*F*_(1,29)_ = 8.159, *p* = 0.008; Figure 4) but no significant interaction, indicating that BCAS and age reduced social interaction to a similar degree.

#### Velocity and Anxiety Behavior in the Open Field (OF) Test

**Figure 5:**
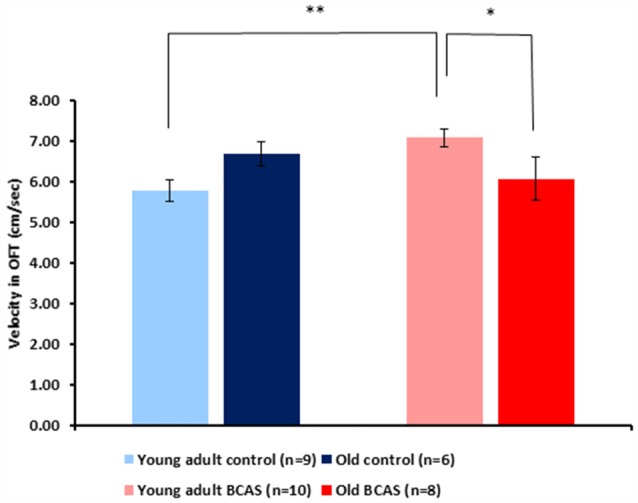
Effect of BCAS microsurgery on velocity of young adult and old mice in the open field (OF) test. BCAS microsurgery induced opposing effects on the velocity of young adult and old mice: it decreased the velocity of the old and increased the velocity of young adult mice. Two-way ANOVA revealed a significant age by treatment interaction (*F*_(1,33)_ = 8.644, *p* = 0.006). Univariate tests of simple main effects indicated higher velocity of young adult BCAS mice compared with both young adult controls (*p* < 0.05) and old BCAS mice (*p* < 0.01; *p < 0.05; **p < 0.01).

BCAS microsurgery increased velocity of young adult and decreased velocity of old mice, reflected by a significant age by treatment interaction (*F*_(1,29)_ = 8.64, *p* = 0.006; Figure 5). Analysis of the amount of time mice spent in the center of the arena showed no significant effect of age or treatment and no age by treatment interaction. Consistent with the main analysis, univariate tests of simple main effect revealed significant difference between old control and old BCAS mice (*F*_(1,29)_ = 5.384, *p* = 0.028), indicating that BCAS microsurgery decreased velocity of old females.

#### Radial-Arm Water Maze (RAWM) Test

**Figure 6:**
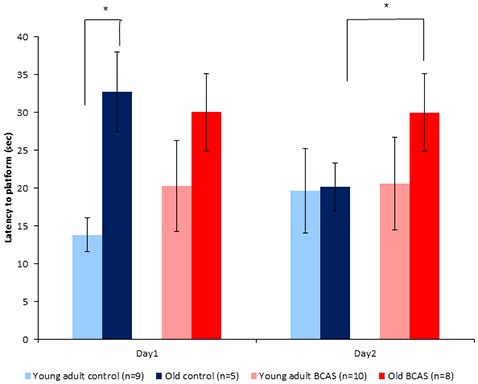
Effect of BCAS microsurgery on latencies to hidden platform of young adult and old mice. Two-way ANOVA with repeated measures revealed an age by time interaction (*F*_(1,29)_ = 7.31, *p* = 0.011) and a triple interaction of age, treatment and time (*F*_(1,29)_ = 6.79, *p* = 0.014). Univariate tests of simple main effects indicated significantly longer latencies of old control mice compared with young adult controls (*p* < 0.05) on day 1. On day 2 *post hoc* comparisons revealed significantly longer latencies of old BCAS compared with young adult BCAS mice (*p* < 0.05; *p < 0.05).

Old mice displayed longer latencies locating the hidden platform on the first day of the RAWM test; two-way ANOVA with repeated measures revealed a significant age by experimental day interaction (*F*_(1,29)_ = 7.31, *p* = 0.011). As can be seen in Figure 6, latencies of old control mice were shortened on day 2, becoming similar to the latencies of young adult mice, whereas latencies of old BCAS mice remained longer, as reflected by triple interaction of age, treatment and experimental day (*F*_(1,29)_ = 6.793, *p* = 0.014). Univariate tests of simple main effects indicated significantly longer latencies of old control mice compared with young adult controls (*F*_(1,29)_ = 7.158, *p* = 0.012) on day 1. On day 2, univariate tests of simple main effects revealed that the latencies of old BCAS mice were significantly longer compared to old control mice (*F*_(1,29)_ = 4.424, *p* = 0.049), whereas latencies of old control mice did not differ from the latencies of young adult control mice.

#### Perceptual Learning in Novel Object Recognition (NOR) Test

A trend towards age by treatment interaction was identified (*F*_(1,29)_ = 3.64, *p* = 0.066), indicating that BCAS microsurgery possibly induced differential effect on young adult and old mice (Supplementary Figure S1). No significant main effects of age and treatment were found when analyzing discrimination rate of the novel object.

### Effect of BCAS on Myelin Breakdown and Microglial Activation in Young Adult and Old Mice

**Figure 7:**
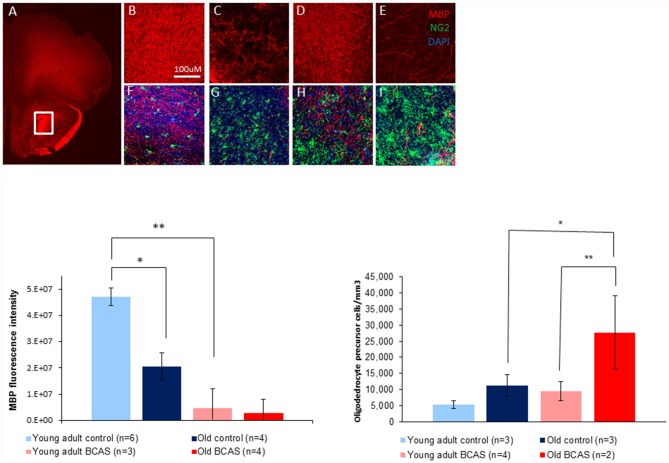
Above: immunofluorescence staining of myelin basic protein (MBP) protein and oligodendrocyte precursors (NG2). Representative picture of anterior commissure area stained for MBP and NG2 **(A)**. Representative pictures of MBP and MBP + NG2 **(B,F)** in young adult control mice, young adult BCAS mice **(C,G)** old control mice **(D,H)** and old BCAS mice **(E,I)** in anterior commissure (Scale bar- 100 μM). Both young adult BCAS mice and old BCAS mice show degradation of white matter in the anterior commissure **(C,E)**. Below Left: MBP fluorescence intensity measurement in the anterior commissure area of young adult control, young adult BCAS, old control and old BCAS mice (×20). Two way ANOVA revealed a significant main effect of BCAS (*F*_(1,13)_ = 11.22, *p* = 0.005) suggesting degradation of white matter in both young adult BCAS and old BCAS groups. Univariate tests of simple main effects indicated a significantly higher fluorescence signal in young adult controls compared with old controls (*p* = 0.04), and young adult BCAS compared with old BCAS (*p* = 0.005; *p < 0.05; **p < 0.01). Below Right: effect of BCAS microsurgery on oligodendrocyte precursor cell density measurements (NG2) in young adult and old mice. Two-way ANOVA revealed significant main effects of age (*F*_(1,8)_ = 6.694, *p* = 0.02) and surgery (*F*_(1,8)_ = 6.190, *p* = 0.038). Univariate tests of simple main effects indicated a significantly higher number of NG2 stained cells in old BCAS mice compared to old controls (*F*_(1,8)_ = 6.694, *p* = 0.032) and compared to young adult BCAS (*F*_(1,8)_ = 9.046, *p* = 0.017).

To determine the effect of BCAS on white matter integrity and inflammation, markers for MBP, oligodendrocyte precursor cells (NG2) and microglia activation (ionized calcium-binding adapter molecule-1 [Iba-1]) were used. As shown in Figure 7 there was degradation of white matter due to both age and surgery. MBP intensity measurement revealed degradation induced by BCAS as reflected by a significant main effect of surgery on two way ANOVA (*F*_(1,13)_ = 11.22, *p* = 0.005). (Figure 7, lower left graph). Univariate tests of simple main effects indicated a significantly higher signal in young adult control mice than in old control mice (*p* = 0.04), and in young adult control mice compared to young adult BCAS mice (*p* = 0.005). It seems that MBP signal is increased in old control mice compared to old BCAS mice, but this result was not statistically significant (*p* = 0.185). These findings suggest that age and hypoperfusion affect white matter intensity in a similar and possibly additive direction.

Oligodendrocyte precursor cell density measurements revealed a higher number of oligodendrocyte precursor cells (NG2 stained) in young adult BCAS and old mice (both control and BCAS) than in young control mice (Figure 7, lower right graph). Two-way ANOVA revealed a significant main effect of age (*F*_(1,8)_ = 8.387, *p* = 0.02) and surgery (*F*_(1,8)_ = 6.190, *p* = 0.038). Univariate tests of simple main effects indicated a significantly higher number of NG2 stained cells in old BCAS mice compared to old controls (*F*_(1,8)_ = 6.694, *p* = 0.032) and compared to young adult BCAS (*F*_(1,8)_ = 9.046, *p* = 0.017).

**Figure 8:**
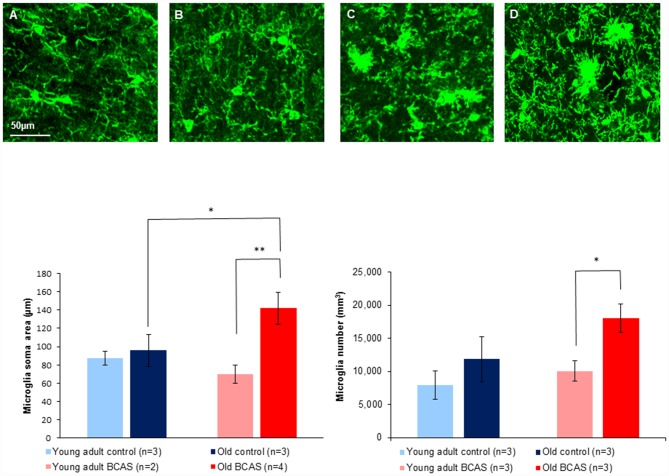
Upper Panel: immunofluorescence staining of microglia (green) in anterior commissure of young adult control **(A)**, young adult BCAS **(B)**, old control **(C)** and old BCAS mice **(D)** (×40 magnification) showing higher level of microglia activation in old BCAS mice in the degraded myelin area than any of the other groups. Lower Panel (left) morphological characterization of microglia revealed that young adult control and BCAS mice had small soma size, suggesting no activation process. Old control and BCAS mice showed a higher level of gliosis (*F*_(1,8)_ = 8.322, *p* = 0.02) reflected by larger soma size . Old BCAS mice exhibited the most substantial activation of microglia (age by treatment interaction *F*_(1,8)_ = 5.283, *p* = 0.051). Univariate tests of simple main effects indicated higher activation of old compared to young adult BCAS mice (*p* < 0.01) and of old BCAS compared with old control mice (*p* < 0.05; *p < 0.05; **p < 0.01). Right: microglia number quantification revealed a higher number of microglia in old mice compared to young adult mice, reflected in main effect of age (*F*_(1,8)_ = 9.242, *p* = 0.016). BCAS microsurgery further increased the number of microglia reflected in a trend towards an effect of treatment (*F*_(1,8)_ = 4.510, *p* = 0.066). Univariate tests of simple main effects further indicated a significantly higher number of microglia in old BCAS mice compared with young BCAS (*p* = 0.021) and marginal elevation compared with old control mice (*p* = 0.056).

Figure 8 (upper panel) shows immunofluorescence staining of microglia (green) in anterior commissure of young adult control (Figure 8A), young adult BCAS (Figure 8B), old control (Figure 8C) and old BCAS mice (Figure 8D). The representative images show a higher level of microglia activation in old BCAS mice (D) in the degraded myelin area than any of the other groups. Microglia were counted in the surface of the anterior commissure, multiplied by the number of stacks and normalized. Soma area and process length of microglia in the upper layer were measured. The left bar graph in Figure 8 shows microglial soma area, which, together with process length, reflects glial activation, in the four experimental groups (young adult control and BCAS, old control and BCAS). Two way ANOVA revealed a significant main effect of age (*F*_(1,8)_ = 8.322, *p* = 0.02) and a marginally significant age by treatment interaction (*F*_(1,8)_ = 5.283, *p* = 0.051). Univariate tests of simple main effects indicated a significantly higher level of microglia activation in old compared to young adult BCAS mice (*p* = 0.007) and in old BCAS compared to old control mice (*p* = 0.032). Microglia number quantification revealed a main effect of age (*F*_(1,8)_ = 9.242, *p* = 0.016) and a trend towards an effect of treatment (*F*_(1,8)_ = 4.510, *p* = 0.066). Univariate tests of simple main effects further indicated a significantly higher number of microglia in old BCAS mice compared with young BCAS (*p* = 0.021) and marginal elevation compared with old control mice (*p* = 0.056). (Figure 8 bar graph right). There were no significant differences between the groups in process.

## Discussion

In this study we examined the effect of cerebral hypoperfusion on behavior and cognition, the integrity of brain white matter and activation of microglia in young adult and aged female mice. Reduced cerebral blood flow was induced by the BCAS procedure whereby stenosis of both common arteries is achieved by means of 0.18 mm surgical steel coils wrapped around both arteries in the course of microsurgery by us as well as by others (Shibata et al., 2004, 2007; Nakaji et al., 2006; Nishio et al., 2010). We demonstrated in a separate group of mice which underwent the operation that the BCAS procedure induces a ~70% reduction in blood flow through the common carotid artery, as measured distal to the coil.

Our aim in the present study was to further explore changes in behavior and cognition induced by BCAS in aged mice and to correlate these with the effects of cerebral hypoperfusion on the brain histochemical measures that we studied. In particular we wished to identify differential effects of cerebral hypoperfusion in aged mice due to the interaction of reduced cerebral blood flow with the effects of age on brain structure and function. Such findings are highly relevant to understanding the pathophysiology of age related brain disorders such as VCI and late life depression which have been linked to impaired cerebral blood flow and brain white matter lesions (Teodorczuk et al., 2007; Brickman et al., 2009; McKinney and Sibille, 2013). Our study demonstrated that BCAS microsurgery had significant effects on social interaction, cognition, myelin density and structure and microglial activation. Cognitive function was differentially impaired by BCAS in aged mice along with more severe white matter lesions and a greater degree of microglia activation in the affected brain areas.

Effects of aging on weight were striking with significant weight loss demonstrated in aged as compared to young adult female mice over the entire 10-week experimental period. The BCAS procedure was not associated with weight loss implying that memory deficits induced by hypoperfusion did not reflect a malnutrition/cachexia-related cognitive phenotype. Although weight loss of aged BCAS mice was higher compared to aged controls, this effect did not reach significance. Similarly we observed an expected effect of age on the generation of new cells in the hippocampal dentate gyrus (Supplementary Data). No significant effect of BCAS was demonstrated on hippocampal neurogenesis, consistent with the findings of Shibata et al. (2004) who found lack of hippocampal damage 30 days after BCAS.

In terms of behavioral effects, BCAS had differential effects on the velocity of young adult and old mice in the OF, increasing the velocity of the young adult and decreasing velocity of the old. This may reflect greater sensitivity of old mice to the impairing effect of BCAS. However, BCAS did not affect the amount of time mice spent in the center of the arena.

BCAS had a significant effect on SE. In both young adult and old mice that underwent the procedure, SE was significantly reduced compared to their counterparts that underwent sham surgery with no coil implantation. There are no previous reports on the effect of BCAS on SE and the current finding is the first to demonstrate an effect of BCAS on an affective behavioral component. However, effects on the Forced swim test (FST) were not significant (Supplementary Data), as in previous reports (Shibata et al., 2007). Moreover, suppressive effects of old age on SE were also demonstrated. The current behavioral studies were performed 8 weeks after BCAS microsurgery. Studies that examine a longer time course are indicated in order to determine whether more pervasive affective findings can be demonstrated. Such findings are of crucial importance in validating BCAS as a model for late life depression.

In contrast to tests reflecting affective components, a unique effect of BCAS was found in aged mice on the radial arm water maze, a spatial test of hippocampal dependent learning and frontal-cortex dependent reference memory. The latencies of old BCAS mice on the second day of the test were significantly longer compared with the latencies of the old controls. The latencies of young adult BCAS mice did not differ from the latencies of young adult controls, implying that cerebral hypoperfusion most severely impairs hippocampal dependent learning and frontal cortex dependent reference memory in aged mice. This finding is consistent with the greater glial activation and higher number of oligodendrocyte precursor cells observed in the frontal cortex of old BCAS mice. A similar trend was found on the NOR test, a measure of perceptual learning. The old BCAS mice displayed the lowest preference towards the new object, while the discrimination rate of young adult BCAS mice was higher. Although not statistically significant, the NOR findings do support the results obtained from the RAWM test, suggesting that BCAS microsurgery induced differential effects on young adult and old mice. As cognitive changes in VCI are much more variable than in other disorders such as Alzheimer’s disease (O’Brien and Thomas, 2015), a consistent phenotype on two distinct cognitive paradigms suggests a biologically-relevant age-dependent differential effect of hypoperfusion.

The translational relevance of the memory-related deficits we observed in aged BCAS mice is bolstered by recent data suggesting that in humans, subtle hippocampal volume and shape changes are evident also in dementias deemed as pure subcortical vascular based on negative 11C-Pittsburg compound-B positron emission tomography scans (Kim et al., 2015). Notably, while such deficits could reflect interruption of corticolimbic or corticostriatal circuits secondary to white matter insults, they could also emanate from subtle hypoperfusion-induced changes to the hippocampus that do not manifest as an overt decrease in neurogenesis. For instance, based on recording of local field potentials, a decrease in synaptic plasticity associated with alteration of information flow has recently been demonstrated in a rat model of VCI (Xu et al., 2013).

Doublecortin (DCX) staining revealed no changes in the number of newly formed neurons in the slices examined from the DG of the hippocampus, between control and BCAS groups (both young adult and old), consistent with the findings of Shibata et al. (2004; Supplementary Data). As opposed to sparing of the gray matter, our MBP and Iba-1 results clearly demonstrate a pronounced effect of BCAS on white matter. Intensity measurement revealed a lower signal of the MBP in both young adult and old BCAS groups, reflected by a significant main effect of treatment. In contrast, white matter of young adult and old control mice seemed intact and well organized in the anterior commissure, thus validating our BCAS model. Notably, in the white matter of young adult BCAS mice there was evidence of minimal damage. Visually, the effect of hypoperfusion was most pronounced in old BCAS mice that showed degradation and disorganization of myelin most pronounced in the anterior commissure area. These findings indicate that on the background of age related changes in the white matter, there is a more pronounced effect of BCAS procedure on old mice than on young adult mice. Given the critical links between white matter integrity and cognitive performance (Arvanitakis et al., 2016), a synergistic detrimental effect of age and hypoperfusion on white matter integrity could underlie the significantly more severe cognitive impairment observed in the old mice that underwent BCAS on the RAWM and NOR tests.

Based on Iba immunostaining of microglia, microglia morphology were apparently normal in young adult mice, both control and BCAS. However, consistent with previous findings (VanGuilder et al., 2011; Hart et al., 2012), we observed a higher level of microgliosis and morphology changes in old mice, both BCAS and control. However, consistent with Coltman et al. (2011) and Saggu et al. (2016), a trend towards an effect of BCAS was observed. Microglia from old BCAS mice exhibited substantial increase in soma size, a morphology change that implies activation of microglia in the degraded myelin area, which, based on two way ANOVA, was significantly higher than all other groups. These striking findings provide further empirical support for previous findings relating to microglial activation in disease and in aging. It has been shown that microglia can become activated or “primed” in chronic neurodegenerative or inflammatory diseases, and these primed cells, in contrast to the normal resident microglia, have a lower threshold for activation and can become harmful upon further stimulation (Cunningham et al., 2009). As the normal aging process can also induce microglia priming (Frank et al., 2010), our data highlight the relevance of the age-by-disease hypothesis, stressing that senescence-related changes in microglial morphology and activity could prime them to be hyperactive upon being exposed to ischemia. Notably, microglia residing within white matter were shown to exist in a relatively less quiescent basal state than their gray matter counterparts (Hart et al., 2012). Hence, an *a priori* more immune-vigilant profile, combined with senescence-related proinflammatory innate immune response (Cribbs et al., 2012), could plausibly be overtly dysregulated when challenged with a severe stressor such as chronic ischemia. Consistent with glial activation observed in old BCAS mice, a significantly higher number of oligodendrocyte precursor cells were visualized in frontal cortex of old BCAS mice compared to young BCAS mice, and also (marginally) compared to old controls. Increased number of oligodendrocyte precursor cells following hypoperfusion is well known (McQueen et al., 2014), and is commonly serves as a measure of myelin degradation (Young et al., 2016). This indeed was also demonstrated by us, reflected in significant effect of treatment. However, two-way ANOVA demonstrated that old BCAS mice displayed the highest rate of oligodendrocyte precursor cells of all groups, implying the most severe myelin degradation.

A link between glial activation and white matter lesions in the BCAS model is well established. Not only was activation of microglia one of the original hallmarks of the BCAS model (Shibata et al., 2004), it was previously demonstrated that inhibiting glial activation decreases damage to the white matter and attenuates the induced cognitive impairments (Lee et al., 2013; Hou et al., 2015; Liu et al., 2015). It has been demonstrated that aging sensitizes microglia to immune challenge (Frank et al., 2010), suggesting that the increased vulnerability of old mice to the deleterious effect of BCAS is at least partly due to increased sensitivity of microglia.

Augmented glial activation and potentiated inflammatory response may serve as a possible mechanism through which BCAS induces greater effect on old mice (Knapp et al., 2016). Indeed, a review by Popa-Wagner et al. (2007a) indicated that hypoxia models of aged rats demonstrate more severe infracts, augmented apoptosis, early activation of macrophages and rapid formation of glial scar tissue. Research conducted by Popa-Wagner et al. (2007b) demonstrated that augmented early inflammatory response may interfere with recovery mechanisms. Stroke models demonstrated more severe neuroinflammation in aged rats (Buga et al., 2013), while oxidative stress induced higher levels of proinflammatory cytokines and chemokines in aged compared with young adult rats (Flowers et al., 2016). Consistently, lipopolysaccharide (LPS) was demonstrated to induce prolonged neuroinflammation and elevated levels of proinflammatory cytokines in aged compared with young adult rats (Fu et al., 2014).

A possible mechanism linking neuronal stress induced by hypoperfusion to glial activation may rely on the mediator fractalkine, a neuronal-derived chemokine, that is known to induced glial activation (Wieseler-Frank et al., 2004; Liu et al., 2015), and release of pro inflammatory cytokines such as IL-1, IL-6, and TNF-alpha (Johnston et al., 2004; Liu et al., 2015). CXCR1 and ERK/p38 MAPK inhibition decreased activation of microglia and blockade of this route also attenuated lesions to the white matter, and the cognitive impairments induced by BCAS (Lee et al., 2013; Liu et al., 2015).

Overall, the results of our studies suggest that aged mice are more vulnerable than young adult mice to the effects of cerebral hypoperfusion. Due to their old age these mice lost weight, displayed decreased SE, and increased RAWM latencies on the first day of the test. They were also characterized by fewer newly formed neurons in the hippocampus, reflected by DCX staining, decreased MBP signal and increased number of microglia compared to their young counterparts. This potential vulnerability may account for the differential effect of BCAS in old mice reflected in impaired cognitive function, more pronounced damage to the myelin sheath at the anterior commissure area, and increased activation of microglia. However, due to the small sample size used in the immunohistochemistry analyses, which is prone to type I and II errors, we regard these results as preliminary. Further studies are required to replicate and validate the present observations.

Taken together, the results of the present study support the hypothesis that vulnerability of the elderly to cerebrovascular white matter lesions that are associated with cognitive impairment and late life depression, may result from elevation of glial sensitivity and high levels of pro-inflammatory cytokines. The use of old mice in combination with relevant experimental procedures offers a powerful tool for better understanding the interaction of aging and brain hypoperfusion and could contribute to the development of novel medications to treat and prevent related disorders.

## Author Contributions

Listed authors contributed to the study and manuscript as follows: GW: experimental design, behavioral experiments, data analysis and manuscript preparation. AL: experimental design, data analysis and manuscript preparation. TL: behavioral experiments and manuscript preparation. HB-A: behavioral experiments, histology and manuscript preparation. TKM: histology and manuscript preparation. MV: blood flow measurements. PT: behavioral experiments and BCAS microsurgery. EA: BCAS microsurgery. BM: behavioral experiment programing. NK: BCAS microsurgery. BL: experimental design, data analysis, manuscript preparation and revision.

## Conflict of Interest Statement

The authors declare that the research was conducted in the absence of any commercial or financial relationships that could be construed as a potential conflict of interest.
