# Supplementary material for: Differentially Severe Cognitive Effects of Compromised Cerebral Blood Flow in Aged Mice: Association with Myelin Degradation and Microglia Activation
Source: Front Aging Neurosci. 2017 Jun 16;9:191. doi: 10.3389/fnagi.2017.00191 (PMC5472721; doi:10.3389/fnagi.2017.00191)
Supplement: Supplementary file 1 [file Data_Sheet_1.docx]

**Supplementary Materials**

**Methods:**

**Forced swim test (FST)**: Mice were placed in a Plexiglas cylinder (21cm diameter) filled with water at room temperature for 6 min test. The amount of time spent swimming compared with floating was calculated. FST was performed using Ethovision 10 system, providing fully computerized, blinded and unbiased measurement. This test was conducted during the 2^nd^ week of behavioral battery.

**Rotarod:** A test of motor abilities, which require mice to balance on a rotating cylinder. The test was consisted on 3 4-minute trail. During each trial rod rotation gradually increased up to 40 rotations/minute. The amount of time mice balanced on the rod in each trail was measured. Trials were divided by at least 20-minute breaks, to avoid mice exhausting. The experimenters were blind to the experimental conditions of each mouse. Rotarod test was conducted during the 2^nd^ week of behavioral battery.

**Doublecortin (DCX) immunofluorescence staining:** DCX staining was performed on frozen floating brain sections. The sections were fixed in methanol, washed twice with PBS and incubated overnight in 1% bovine serum and 0.1% triton in 1XPBS with the primary antibody (Anti-Doublecortin -1 1:3000, Millipore-Temecula, CA, USA) at 4 ^0^C. Sections were then incubated with the secondary antibody (Cy5, donkey anti guinea pig, 1:400; Jackson ImmunoResearch) for 2 h at room temperature (RT) and counter-stained with DAPI (Sigma, Israel). DCX images were captured using an Olympus FV-1000 confocal microscope and camera (Tokyo, Japan). The number of DCX marked cells was manually counted at x20 in a defined area containing the entire granular cell layer (GCL) of the hippocampus. DCX marked cells were counted twice by an observer blind to age and exposure. Number of visible DCX-stained cells was divided by the GCL volume. Volume of GCL was calculated as following: (GCL area * number of stacks * stack spacing * 0.621^2^ )/ 10^9^.

**Results:**

**Depressive phenotype in forced swim test (FST):**

No significant age (F[1,29]=1.109, p=0.301), treatment (F[1,29]=0.28, p=0.868), or age by treatment interaction (F[1,29]=0.74, p=0.787) were demonstrated.

**Motor activity in rotarod test:** Young adult mice displayed longer latencies in the rotarod test, reflected by trial by age interaction (F [2,52]=5.22, p=0.009). No significant interactions of trial and treatment (F[2,52]=0.14, p=0.986) or trial, age and treatment (F[2,52]=0.741, p=0.481) were demonstrated.

**Effect of BCAS on neurogenesis:** As shown in **Supplementary** **Fig. 2a-d**, by immunofluorescence staining with doublecortin (DCX), there was a significantly lower number of newly formed neurons in the slices examined from the dentate gyrus of the hippocampus in old as compared to young adult female mice across both BCAS and control groups (F [1,15]=92.60, p=8*10^-8^). Univariate tests of simple main effects showed a significant difference between young adult (n=5) and old (n=4) control mice (p=2*10^-5^) and between young adult (n=6) and old (n=4) mice that underwent BCAS microsurgery (p=10^-6^). However, there was no significant effect of BCAS in either age group and no age by treatment interaction.


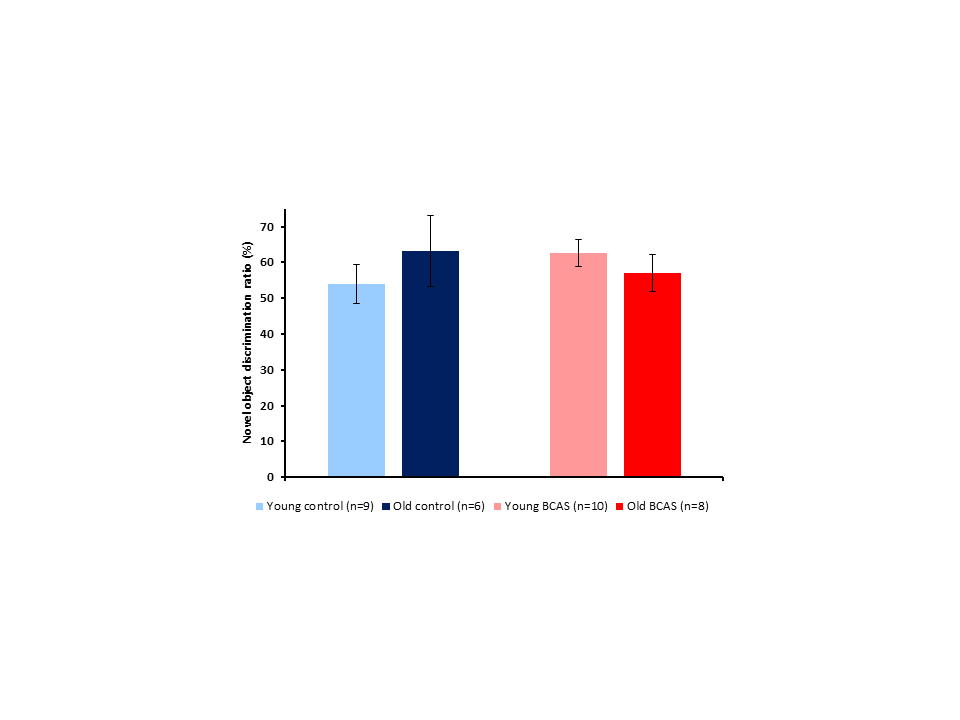


**Supplementary Fig. 1.** Effect of BCAS on perceptual learning of old and young adult mice in the NOR test. A trend towards age by treatment interaction was identified (F [1,29]=3.64, p=0.066), indicating that BCAS microsurgery possibly induced differential effect on young adult and old mice. No significant main effects of age and treatment were found when analyzing discrimination rate of the novel object.


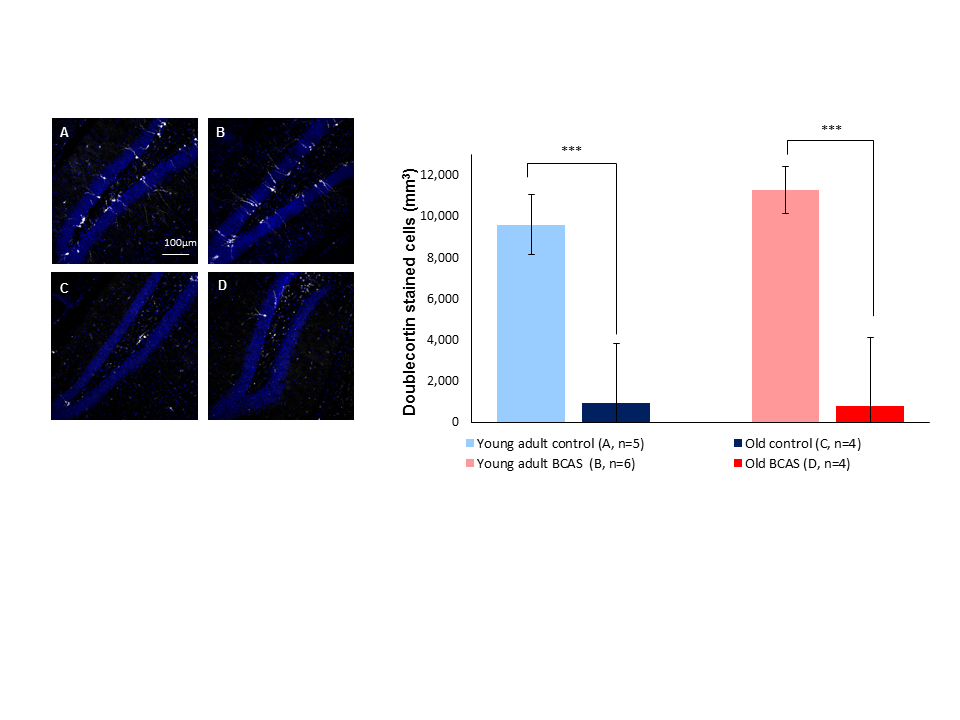


**Supplementary Fig. 2.** Immunofluorescence staining of doublecortin (DCX) in the granule cell layer of the dentate gyrus of hippocampi of young adult (3 months) control (a, n=5) and BCAS (b, n=6) female mice, and old (21 months) control (c, n=4) and BCAS (d, n=4) female mice. Two-way ANOVA revealed a significant main effect of age (F [1,15] = 92.61, p=8*10^-9^) on the number of DCX stained cells. Using univariate tests of simple main effects a significant difference was found between young adult (n=5) and old (n=4) control mice (***p<0.001) and between young adult (n=6) and old (n=4) BCAS mice (***p<0.001).
